# Comparison of Red Blood Cell (RBC) Histogram and Indices on Peripheral Blood Smears for the Detection of Anemia: A Study Protocol

**DOI:** 10.7759/cureus.69197

**Published:** 2024-09-11

**Authors:** Vaishnavi Rajurkar, Lokesh Singh Chauhan

**Affiliations:** 1 Pathology, School of Allied Health Sciences, Datta Meghe Institute of Higher Education and Research, Wardha, IND; 2 Pathology, Jawaharlal Nehru Medical College, Datta Meghe Institute of Higher Education and Research, Wardha, IND

**Keywords:** anemia, hemoglobin, normocytic, peripheral blood smear, rbc histogram

## Abstract

Background

Anemia is characterized by a lower red blood cell (RBC) count than the usual rate or the average count that should be found in the blood. It is described by the reduction in the concentration of hemoglobin, the number of RBCs, and the O2-carrying capacity of the blood. A complete blood count (CBC) gives serious information on changes in the size and shape of RBCs and an indication of inclusion bodies that will help exclude anemia. RBC indices suggest potential reasons for the anemia in a particular patient. Thus, RBC indices can give some clues about the possible cause of anemia in a given patient with the disease. It is also necessary to look at the peripheral blood smear (PBS) and RBC indices and histogram to evaluate anemia adequately.

Method

The primary complaints of volunteers, such as clinical anemia as well as essential family medical history, will be the criteria for selection. After that, the main procedures that will be performed are the PBS and CBC. The blood sample will be drawn while the individual sits securely and in an aseptic setting. An ethylenediaminetetraacetic acid (EDTA) tube will be used to collect 2 ml of venous blood, which will then be gently mixed and processed on a five-part automated hematology analyzer (Coulter) for CBC test and PBS.

Expected results

The diagnostic capabilities of the PBS procedure are expected to match with RBC histograms and indices in the CBC report for the detection of anemia. Comparing both results with the PBS evaluated at the microscopic assessment, it is expected to likely provide a more precise and quantifiable result on RBC morphology and indices with greater accuracy for anemia detection in accordance with the PBS report. Implementing RBC histogram analysis in routine clinical practice is expected to enhance the diagnostic processes for anemia, leading to a more effective and timelier patient treatment.

Conclusion

These findings will suggest that applying RBC histograms and indices in CBC reports in supportive diagnosis with PBS analysis in the everyday clinical practice of hematology may improve the diagnoses and overall care of patients with anemia. Advanced research should confirm the efficacy of integrated approaches to increase diagnostic accuracy.

## Introduction

Based on the study across the world, 40% of children aged 6-59 months, 37% of pregnant women, and 30% of women in the age group of 15-49 years suffer from anemia [1]. By using the WHO criteria, severe anemia refers to hemoglobin (Hb) levels less than 70 g/L among children aged below five years and less than 80 g/L among other age groups [2]. This ensures that treatment is tailored to address the root cause, whether it be nutritional deficiencies, chronic diseases, or genetic disorders, leading to better patient outcomes. Abnormalities in the size, shape, or color of red blood cells (RBCs) (anisocytosis, poikilocytosis, hypochromia) are valuable assets in the distinction between the various types of anemia. For example, microcytic and hypochromic RBCs are characteristics of iron deficiency anemia, while macrocytic RBCs may suggest malnutrition and vitamin B12 or folate deficiency [3].

Anemia is diagnosed using the peripheral blood smear (PBS), where a microscopic analysis of the blood smear provides valuable knowledge about the variation of the shape and size of RBCs or the presence of any inclusion bodies. Microscopic examination can show an abnormality in the size and shape of RBCs when analyzing a smear of the peripheral blood to identify anemia [4]. Morphologically, anemia can be categorized using RBC indices [5]. RBC indices derived from RBC and Hb analysis are essential in determining the underlying cause of anemia. Using such indices, it also becomes possible to distinguish between microcytic, macrocytic, or normocytic anemia [6]. RBC indices measure the size and concentration of Hb in RBCs. Irregular results affect the type and concentration of anemia [7]. The mean corpuscular volume (MCV) is the sample's average quantity of all the counted RBCs. The value is expressed in terms of femtoliters (fL), a volume unit of measurement, and it typically ranges from 80 to 100. The mean weight of Hb per cell is known as the mean corpuscular hemoglobin (MCH). It usually ranges from 27 to 33 picograms. The mean corpuscular hemoglobin concentration (MCHC) represents the average weight of Hb per cell relative to its volume. It typically has a value of 32-36 g/dL [8].

When an erythrocyte's average size is lower than usual, it is known as microcytic anemia (MCV <80 fL). Generally observed in long-term iron deficiency anemia, a variety of conditions can induce macrocytic anemia, a deficiency of vitamin B12, which is characterized by a mean RBC volume that is more than usual (MCV >100 fL). Normocytic anemia is defined as anemia with a low blood count but an MCV in a typical range of 80-100 fL. It can be caused by hemolytic anemia, dietary deficiencies, or renal failure [9].

An automated hematology analyzer produces a graphical representation known as an RBC histogram. It is a standard part of a complete blood picture, which supplies clues in the diagnosis of various RBC disorders and gives valuable data regarding RBC parameters like red blood cell distribution width (RDW), MCH, and MCV [10,11]. The graphical representation of cell frequencies versus size is called a histogram. A cell's size is displayed on the x-axis, while the overall number of cells is displayed on the y-axis. They provide crucial guidance for diagnosing and managing severe disorders affecting RBC [12]. PBS examination, RBC histograms, and RBC indices are essential tools for accurately diagnosing and classifying anemia [13].

## Materials and methods

Study design**

This cross-sectional study was carried out in the Central Laboratory Department of Pathology at Acharya Vinoba Bhave Rural Hospital, affiliated with Datta Meghe Institute of Higher Education and Research (Deemed to be a University), located in Sawangi, Wardha, Maharashtra, India. The study commenced following the receipt of participants' consent and has been approved by the Institutional Ethics Committee of Datta Meghe Institute of Higher Education and Research (approval number: DMIHER (DU)/IEC/2024/157). It was also registered with the Clinical Trials Registry of India (CTRI/2024/04/066473) (Appendices). A minimum of 314 eligible participants were involved, and the study spanned one year to ensure comprehensive data collection.

Participants

Patients of Indian origin aged 11-65 years diagnosed with anemia will be included. Exclusion criteria include patients diagnosed with leukemia, lymphoma, or other blood disorders as well as those under 11 or over 65 years of age. Patients with chronic kidney disease and liver disease will be excluded as these diseases affect the production of RBS and the indices of erythropoiesis. Patients who are on chronic medications which are known to alter the morphology of RBCs including chemotherapy will also be excluded. Those patients, who were transfused with blood within the last three months, will be excluded from the study since it may shift their RBC parameters slightly.

Data collection procedure

Both the benefits and drawbacks of their participation will be discussed with the participants. To avoid any misunderstandings, participants will be given written information about the consent before their enrolment. Potential volunteers will be chosen based on their primary complaints such as clinical anemia and family history. The blood sample will be drawn while the individual sits securely and in an aseptic setting. Two milliliters of venous blood will be collected in an ethylenediaminetetraacetic acid (EDTA) tube, gently mixed, and processed using a five-part automated hematology analyzer (Coulter) for complete blood count (CBC) testing and the estimation of RBC indices, including MCV, MCH, MCHC, and RDW. Based on various RBC indices and a histogram, the Beckman counter-analysis report will be examined to diagnose the kind of anemia. Simultaneously, a blood smear will be prepared in a Blue Star slide (75 mm length, 25 mm width, 1.35 mm thickness) from the given blood sample and allowed to dry. After that, staining will be performed using Leishman's stain and Leishman's buffer. The smear will next be examined under a microscope with an oil immersion lens (100×), and the findings will be recorded to make the morphological diagnosis of anemia. One pathologist will independently perform the morphological analysis to ensure accuracy and consistency. The PBS and cell counter methods will be used to collect the data, and the data will be compared and analyzed by using tests like the paired t-test and chi-squared test to draw a conclusion.

CBC

After the blood sample is collected in the EDTA tube, it will be sent to the pathology processing lab. The sample will be aligned in a cassette for processing. Then the cassette will be kept in the Beckman Coulter B1 (UniCel DxH 800). By using the principle of electrical impedance, results will be given by the machine, and this result will be used to analyze CBC and its relation with anemia. Based on various RBC indices and a histogram, the Beckman counter-analysis report will be examined to diagnose the kind of anemia.

PBS

PBS provides valuable information for diagnosing anemia by examining minute variations in the morphology of blood cells. The procedure is as follows: A small layer of blood will form on the smear. After allowing it to air-dry, place them on a dyeing rack. After pouring Leishman's dye drop by drop on the slide, set it for two minutes. This makes it possible to calculate peripheral blood film (PBF) in methyl alcohol. After pouring twice as much buffering water on the slide by drops, let it rest for eight minutes before washing it for a minute or two. After allowing it to air-dry, examine it using a microscope's oil immersion lens. After that, the smear will be examined under a microscope with an oil immersion lens (100×) to determine the morphological diagnosis of anemia, and the results will be recorded. Abnormalities in the size, shape, or color of RBCs (anisocytosis, poikilocytosis, hypochromia) are valuable assets in the distinction between the various types of anemia.

Sample size

The sample size was calculated using Cochran's formula by considering the proportion of the estimated prevalence of anemia (p=28.6%) from the reference article and the estimation error (d = 5%); hence, calculating the total number of anemic patients visiting the hospital, the population size (N) of 314 patients is needed in the study.

The sample size for this study was determined using Cochran's formula. The formula used is as follows: n>=𝑍²1-𝛼/₂×(1−𝑝)/E2. Here, 𝑍²1-𝛼/₂ is the level of significance at 5%, i.e., confidence interval is equal to 1.96, p is equal to 28.6% or 0.286, and E (error of margin) is equal to 5% or 0.05. Thus, n>=1.96^2^×0.286×(1−0.286)/(0.05)², resulting in 313.7 or 314 patients needed in the study [14].

Outcome measures

Under a microscope, we will examine the RBCs in anemic patients for abnormalities such as variations in size, shape, and color, which are indicative of different types of anemia. However, CBC, which reports the amount of Hb in the blood, is always helpful for diagnosing anemia.

Statistical analysis

The complete analysis dataset will consist of all the anemic patients with no missing values for any parameters in the dataset. The primary variables include MCHC, MCV, RDW, and MCHC. All the data will be summarized for the PBS and automated hematology analyzer results of the anemic patients. Demographic variables will be presented using frequencies and percentages for categorical data and means and standard deviations for continuous data. Outcome variables for continuous data will be analyzed and summarized with maximum, mean, minimum, standard deviation, standard error, and a 95% confidence interval for parametric data. Continuous outcome variables will first be tested for normality using the Kolmogorov-Smirnov test at a 5% significance level (p≤0.05). To compare the results of CBC and PBF methods, data will be evaluated finding the sensitivity, specificity, positive predictive value (PPV), negative predictive value (NPV), and accuracy for both methods. If the data is normally distributed, a paired t-test will compare the means of related groups (CBC group with positive and negative cases). If the data is not normally distributed, the Mann-Whitney test will be used. However, the PBS group with positive and negative cases will be evaluated using the chi-squared test or McNemar's test. This analysis will determine the more effective diagnostic method.

## Results

This study therefore seeks to determine whether an RBC histogram can diagnose the three types of anemia, microcytic, macrocytic, and normocytic [15], with reference to the traditional method of diagnosing the condition using the PBS examination. This study will determine the efficiency of RBC histogram analysis by comparing the diagnostic findings with that of PBS examination which will be used. Thus, the use of this approach will offer the researcher an understanding of how efficient RBC histogram analysis is as compared to the PBS examination in the identification of diverse types of anemia. Better results in diagnostic capabilities are expected, with RBC histograms likely providing more precise and quantifiable data on RBC indices. Implementing RBC histogram analysis in routine clinical practice is expected to enhance diagnostic processes for anemia, leading to a more effective and timelier patient treatment.

Table 1 shows the metric table to compare the RBC histogram and PBS.

**Table 1:** Metric table to compare the RBC histogram and PBS RBC: red blood cell; PBS: peripheral blood smear; PPV: positive predictive value; NPV: negative predictive value

Metric	RBC histogram	PBS
Sensitivity		
Specificity		
PPV		
NPV		
Diagnostic efficiency (overall accuracy)		
Time per sample		
Cost per test		

## Discussion

However, the reliance on manual examination in PBS introduces potential variability and subjectivity. The accuracy of PBS largely depends on the skill and experience of the pathologist. Additionally, PBS can be time-consuming and labor-intensive, which may delay diagnosis and subsequent treatment. In contrast, RBC histograms and indices provided by automated hematology analyzers offer a more standardized and quantitative approach. RBC indices, such as MCV, MCH, MCHC, and RDW, are essential for classifying anemia and understanding its underlying causes. For example, low MCV and MCH values suggest microcytic anemia, while high MCV indicates macrocytic anemia. These indices can also help identify mixed anemia cases where different types coexist [13]. RBC histograms are essentially a graphical illustration of the distribution of RBC sizes and enable better predictions of the heterogeneity of the cell population. Another advantage of such graphical data is that PBS scattering patterns that may not reveal perceivable abnormalities to the naked eye may distinctly appear contrasting when represented graphically. Moreover, automated analysis lowers the possibility of human inaccuracy and increases the likelihood of a quality study repeating. In as much as automated analyzers come with these benefits, they have also been known to possess the following disadvantages. They can miss some morphological changes or peculiar diseases that a skilled pathologist is capable of diagnosing with the help of PBS. Furthermore, the expense of automatic machinery and the services required to maintain them make them almost impossible for several healthcare units particularly in developing nations [15]. The study aimed to compare the diagnostic outcomes of two methods, PBS examination and automated cell counter parameters, to identify any differences or similarities and revealed that histograms are vital for initial blood sample analysis, providing insights into RBC count, MCV, and RDW through their shape and shifts. They can hint at potential pathologies and guide the need for detailed PBS examination. While automated analyzers reduce workload with graphical data, our study emphasizes the importance of confirming these results with microscopy for accuracy [16].

The PBS examination is crucial for diagnosing hematological disorders like anemia. Despite its accuracy, manual PBS analysis remains tedious, error-prone, time-consuming, and reliant on skilled laboratorians. While manual microscopic evaluation is the gold standard for PBS analysis, an automated system is crucial for quicker, more accurate diagnosis. The review highlights the need for a large, annotated PBS dataset and a robust system to handle staining and imaging variations. A hybrid method combining morphological and clinical features could enhance anemia classification efficiency [4]. This study examined the relationship between RBC histograms from the Abbott Cell-Dyn Ruby analyzer and PBS results in 500 blood samples. Dimorphic anemia cases showed normal MCV, MCH, and MCHC, with increased RDW due to anisocytosis and poikilocytosis. Macrocytic anemia cases had elevated MCV, MCH, and RDW with normal MCHC. The study concluded that histograms complement PBS in diagnosing RBC disorders and reliably correlate with PS interpretations in anemia cases [17]. In another study of 354 adult blood samples with Hb <10 mg/dl, RBC histograms and PBS were compared to classify anemia into four types. The majority of cases were normocytic, with histograms showing high sensitivity for normocytic and macrocytic anemia but low specificity. Microcytic anemia had higher specificity but lower sensitivity. Overall, histograms were effective for detecting normocytic and microcytic anemia. Discordance in macrocytic anemia was noted due to histograms detecting subtle morphological changes not visible under light microscopy [18]. This study expects that the integration approach of these two diagnostic procedures will give a broader view and understanding of anemia. Clinicians get a greater accuracy by the cross-verification of results of RBC histograms and indices with PBS findings. For example, automated analysis can quickly and accurately look through and differentiate between the types of anemia, and PBS can then offer confirmation of the findings or point out the abnormalities that the automated system did not pick up on. Also, integrating RBC histogram analysis in the daily practice of diagnosis may reduce the time taken in diagnosis and increase the speed at which they are conducted. This can result in faster actions by the healthcare provider and improved care for the patient, as prompt identification of anemia is key to treating it. The study results are expected to provide valuable insights into the integration of these diagnostic tools, enabling them to function cohesively within a unified system. The identification of the analyzers offers remarkable precision, while the PBS provides detailed morphological insights [4].

Limitations

Lack of Morphological Detail

RBC histograms do not provide information on cell shape or other morphological abnormalities, which are often crucial for diagnosing conditions like spherocytosis or sickle cell anemia. This can necessitate additional testing with a PBS or other diagnostic methods.

Calibration Dependency

The accuracy of RBC histograms is highly dependent on the proper calibration and maintenance of the hematology analyzer. Calibration drift can lead to erroneous results, which is less of a concern with manual PBS.

## Conclusions

Based on these observations, the reporting of RBC histogram analysis and indices together with the conventional CBC along with PBS might expand the diagnostic yield for several types of anemia towards routine hematology practice. It is therefore possible that this integrated approach might assist in the better distinction between the causes of anemia and in the further delivery of quantitative and qualitative management of the condition. Therefore, by using hematology analyzers in conjunction with conventional morphological methods, the quality of care given to patients with anemia can be improved. Future studies should replicate these findings in bigger sample sizes and look into more sophisticated technologies like artificial intelligence to enhance the diagnostic specificity and ensure that patients are given the best diagnosis and subsequently the best treatment.

## Figures and Tables

**Figure 1:**
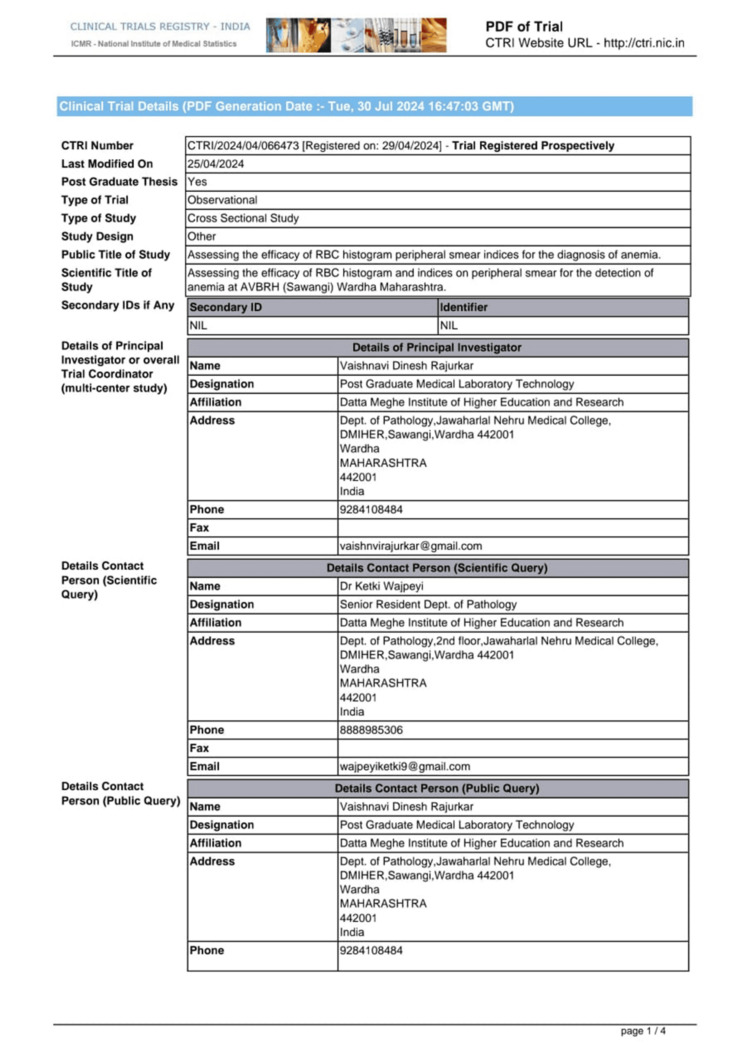
CTRI registration CTRI: Clinical Trials Registry of India
